# Dietary Arginine Supplementation Modulates the Proteome of Boar Seminal Plasma

**DOI:** 10.3390/ani15040555

**Published:** 2025-02-14

**Authors:** Emmanuel O. Oladejo, Tasha R. Gruhot, Seongbin Park, Ghassan M. Ishak, Benny E. Mote, Shengfa F. Liao, Jean M. Feugang

**Affiliations:** 1 Department of Animal and Dairy Sciences, Mississippi State University, Mississippi State, MS 39762, USA; eoo33@msstate.edu (E.O.O.); sp1679@msstate.edu (S.P.); s.liao@msstate.edu (S.F.L.); 2 Department of Animal Science, University of Nebraska-Lincoln, Lincoln, NE 68583, USA; trgruhot@gmail.com (T.R.G.); benny.mote@unl.edu (B.E.M.); 3 School of Medicine, Southern Illinois University, Carbondale, IL 62901, USA; ghassan@siu.edu

**Keywords:** arginine, boar, seminal plasma, spermatozoa, proteome

## Abstract

This study investigated the impact of an increased arginine (ARG) level in a boar diet on semen production, sperm quality, and seminal plasma proteome. Adult Nebraska Index Line boars were assigned to two groups, one receiving a control diet with 0.77% arginine (*n* = 4) and the other a high-arginine diet with 1.77% arginine *(n* = 5). Semen was collected twice a week over the whole experiment, including one week before, six weeks during, and six weeks after the supplementation. Parameters such as semen volume and concentration were assessed immediately after collection, alongside sperm motility and morphology. Centrifugation of raw semen samples yielded seminal plasma for a gel-based proteome analysis. The seminal plasma proteins were extracted, quantified, and separated via 2D gel electrophoresis, allowing protein identification through mass spectrometry. Data analysis involved two-way ANOVA for comparisons (*p* < 0.05). Results showed that arginine supplementation improved semen volume and total sperm counts, with averages of 21 ± 3 doses in the control group versus 24 ± 2 in the ARG group (*p* = 0.05). Although sperm motility and morphology remained unaffected (*p* > 0.05), dietary arginine upregulated ten proteins and downregulated two. In summary, increased dietary arginine did not significantly alter key parameters of semen output or sperm quality but significantly impacted seminal plasma proteome, warranting further research on sperm viability.

## Simple Summary

This study explores the effects of an increased arginine level in a boar diet on semen production and the seminal plasma proteome. The results showed that although arginine supplementation did not significantly affect semen output or sperm quality, such as in motility and morphology, it led to an increase in ten proteins and a decrease in two proteins related to reproduction. These findings suggest that arginine may influence sperm function, highlighting the need for further research in this critical area.

## 1. Introduction

Efforts to enhance fertility and improve livestock production are crucial as the rising demand for meat, dairy, and other animal products to meet the nutritional needs of the growing population remains unmet [1]. This challenge is intensified by global warming, which negatively impacts animal spermatogenesis and, in turn, fertility [2,3,4,5,6]. Improving fertility in livestock, mainly swine, which accounts for 40.4% of global meat consumption [7], is vital for advancing animal reproductive efficiency and addressing the increasing food demands. It has been known that nutritional strategies can present a cost-effective approach to enhancing reproductive efficiency in mammals [8,9,10,11]. Research has shown that dietary L-arginine (commonly known as arginine) supplementation could significantly boost male fertility and reproductive performance [12,13]. This is due to its versatile role in the animal body, which influences various physiological functions, including some spermic functions [14,15]. Nonetheless, the effect of the highest doses necessary to determine the optimal arginine range in boosting male fertility, especially in boars, is still unknown or reported.

Arginine is a crucial amino acid precursor for producing nitric oxide through an enzyme called nitric oxide synthase, which is vital for sperm cellular functions [14,15]. Research in boars has shown that dietary arginine (Arg) supplementation enhanced insulin sensitivity [16] and significantly altered body metabolic profiles [17]. Additionally, Tan et al. [18] found that arginine increased the expression of genes responsible for lipogenesis in skeletal muscle and lipolysis in white adipose tissue. Dietary arginine supplementation for gilts during days 30 to 114 of gestation improved fetal survival rates and increased piglet birth weights [19]. Also, Arg has been shown to enhance antioxidant capacity while reducing superoxide release [20]. All these findings make a strong case for incorporating Arg into dietary strategies for better health and metabolic performance of pigs. On the other hand, some studies show no or adverse effects of different L-arginine levels in pregnant sows’ diets [21,22].

Dietary L-arginine supplementation significantly benefits male fertility in various species [23,24], including pigs [12]. These studies indicated arginine’s beneficial effects in boosting libido, testosterone level, semen production, and sperm quality. Yet, little is known regarding arginine’s impact on the seminal plasma, the nutritive and protective microenvironment of the spermatozoon. In pig nutrition, maintaining body weight and normal physiological functions takes place around 0.5–1.0% Arg, mainly observed in growing, pregnant, and lactating pigs [25]. Therefore, we hypothesized that higher arginine levels (1.77%), beyond maintenance requirements (e.g., 0.77%), could benefit boar reproductive performance due to its role in nitric oxide production and spermatogenesis. The mechanisms by which dietary L-arginine supplementation enhances semen production and quality are not fully understood. However, seminal arginine availability, influenced by nutrient intake and affecting spermatogenesis [26], is essential for fertility due to its direct action in stabilizing sperm DNA packaging [27,28,29]. Meanwhile, its indirect action through protein synthesis remains unexplored. Therefore, the current study conducted proteomic analyses to assess how a higher dietary L-arginine level affects the boar seminal plasma proteome using bioinformatics to predict its impact on sperm fertilization potential.

## 2. Materials and Methods

### 2.1. Animals, Diets, and Feed Management

Ten adult boars (~20 months of age) from generation 35 of the Nebraska Index Line [30] were individually housed in a ventilated barn with a controlled temperature of about 20 °C at night and 26 °C during the day at the University of Nebraska Lincoln (UNL) Swine Research farm. The experiment was conducted from 31 July to 6 November, and boars were fed twice (7:00 h and 14:00 h) daily with a total of 1.8 kg of a fortified corn and soybean meal-based diet. The boars were randomly allocated into two groups, either a basal diet (CON; *n* = 5) or the treatment diet (supplemented with 1% L-arginine; ARG, *n* = 5). L-arginine was added to the ARG diet, topping it up to 1.77%, from 0.77% in the basal diet. The boars had free access to water across the three periods of the experiment: two-week pre-treatment (Pre-ARG), six-week arginine treatment (ARG), and six weeks post-treatment (Post-ARG).

### 2.2. Boar Semen Collection, Semen Outputs, and Sperm Analysis

The boars were trained for semen collection from a phantom mount, and semen samples were collected by a skilled technician using an artificial vagina. Semen collections started from July to November 2017 and were performed twice a week (Monday and Thursday) during the three periods of the experiment (Pre-ARG, ARG, and Post-ARG). The volume of semen was evaluated (estimated by weighing it using the conversion of 1 g of semen equal to 1 mL of semen), and the concentration was assessed using a self-calibrating photometer (SpermCue, Minitube of America, Verona, WI, USA). Semen samples were mixed with a commercial extender (PRIMXcell; IMV Biotech, Inc., Maple Grove, MN, USA). Sperm motility and morphology were measured at 37 °C in duplicates using a computer-assisted sperm analyzer (CASA; Hamilton-Thorne Sperm Analyzer IVOS 1.9, Hamilton Thorne Biosciences, Beverly, MA, USA). Various motion (i.e., total motility), morphology (i.e., abnormalities), and velocity (i.e., average path or VAP, curvilinear or VCL, and straight-line or VSL) parameters were recorded from the analysis.

### 2.3. Seminal Plasma Collection and Protein Extraction

After collection, aliquots of raw semen were subjected to a 650× *g* centrifugation for 10 min at 4 °C to pellet spermatozoa and other contaminant cells. The supernatants were collected and subjected to another centrifugation at 12,000× *g* for 15 min to eliminate additional sperm pellets. The supernatant, seminal plasma free of spermatozoa and cellular debris, was carefully removed, and aliquots were used for total protein analysis using a Pierce Coomassie Plus assay kit (Thermofisher, Waltham, MA, USA). The remaining samples were stored at −80 °C for proteomic analysis.

### 2.4. Seminal Plasma Protein Extraction and Proteomic Analysis

This experiment was conducted on samples taken after a six-week treatment, comparing the control group (CON, *n* = 4) with the arginine group (ARG, *n* = 5). This methodology enables an evaluation of the effects of arginine over the entire duration of spermatogenesis, which includes the complete cycle of sperm production. Total protein was precipitated from the frozen-thawed samples by adding 25% trichloroacetic acid (TCA) solution for 24 h. The precipitated protein was washed with cold acetone three times, and protein pellets were dissolved in the rehydration sample buffer [7 M urea, 2 M thiourea, 4% CHAPS (*w*/*v*), and 20 mM dithiothreitol]. After protein quantification, 500 µg were loaded onto the isoelectric focusing (IEF) strips (ReadyStrip IPG strips, 11 cm, pH 3–10, Bio-Rad Laboratories, Hercules, CA, USA). The IEF electrophoresis was carried out with conditions of 30 V for 12 h (rehydration), 500 V for 15 min, and 8000 V for 2.5 h, with a total of 35 kVh. After IEF, the strips were incubated for 15 min in equilibration buffer (6 M urea, 20% glycerol, 2% SDS, and 0.375 M Tris-HCL pH 6.8) containing 2% dithiothreitol (*w*/*v*), followed by additional incubation for 15 min in equilibration buffer containing 2.5% idoacetamide (*w*/*v*). Electrophoresis was then carried out by transferring the strips onto 4–20% gradient pre-casting gels (Criterion TGX Precast Gels 4–20%, Bio-Rad). Thereafter, the gels were stained with Bio-safe Coomassie G-250 solution (Bio-Rad). Three independent biological replicate gels per sample experimental group (CON and ARG) were obtained for scanning using Proteome Works Plus Spot Cutter (Bio-Rad). All images were aligned, and protein spots were detected by PDQuest 2-D analysis software (v7; Bio-Rad). Each protein spot was normalized based on the total valid spot intensity, and all detected spots were quantified for statistical analysis. Spots of significant difference showing neat separation and/or higher protein amounts were selected and extracted for further protein identification using matrix-assisted laser desorption ionization-time of flight/time of MALDI-TOF/TOF mass spectrometry (MS).

### 2.5. In-Gel Digestion, MALDI-TOF/TOF, and Functional Analyses

In-gel tryptic digestion and subsequent MS spectra analysis were conducted at the Institute for Genomics, Biocomputing, and Biotechnology (IGBB), Mississippi State University. Briefly, the protein spots showing significant differences between the CON and ARG groups were automatically excised from the gels. Digested and dried peptides were dissolved in 2 μL of a saturated solution of α-cyano-4-hydroxycinnamic acid in 50% acetonitrile and 0.1% of trifluoroacetic acid. As the internal standards, des-Arg-bradykinin (monoisotopic mass, 904.4681) and angiotensin I (1296.6853) were mixed with dissolved peptide samples loaded onto MALDI target plates. Spotted peptide samples were analyzed by MALDI-TOF/TOF MS (4700 Proteomics Analyzer, Applied Biosystems, Waltham, MA, USA). Monoisotopic peptide masses were selected from the 800 to 2500 Da mass range with an acceleration voltage of 20 KV. The mass spectra were acquired by a cumulative average of 300 laser pulses, and protein identification was performed by peptide mass fingerprinting (MASCOT: http://www.matrixscience.com/server.html (accessed on 15 December 2020) with a mass tolerance of ±50 ppm. The classification of biological processes and functional annotation of those identified proteins were performed using the COG (Clusters of Orthologous Group) analysis (https://www.ncbi.nlm.nih.gov/research/cog; searched on 15 August 2023). In addition, protein interaction analysis was performed using STRING v10.5 (https://string-db.org/cgi/input?sessionId=bjTuB1Xs3Rrt&input_page; searched on 1 September 2023).

### 2.6. Statistical Analysis

The effects of Arg were evaluated using the general linear model procedure (PROC GLM) of SAS (version 9.4; SAS Institute Inc., Cary, NC, USA). The semen data were analyzed with a two-way analysis of variance (ANOVA-2), followed by pairwise comparisons between treatment periods (Pre-ARG, ARG, and Post-ARG groups). Additionally, repeated-measures analysis was used to test for a fixed effect or two-way interaction of treatment, week, and treatment periods. The normality of data was evaluated using the Shapiro–Wilk test, with the square root transformation used for normalization when needed. Data were presented as mean ± standard error of the mean (sem). For proteomics analysis, elected protein spots were compared with Student’s *t*-test to determine downregulated and upregulated proteins (*p* < 0.05).

## 3. Results

### 3.1. Boar Semen Production and Sperm Motility

**Figure 1:**
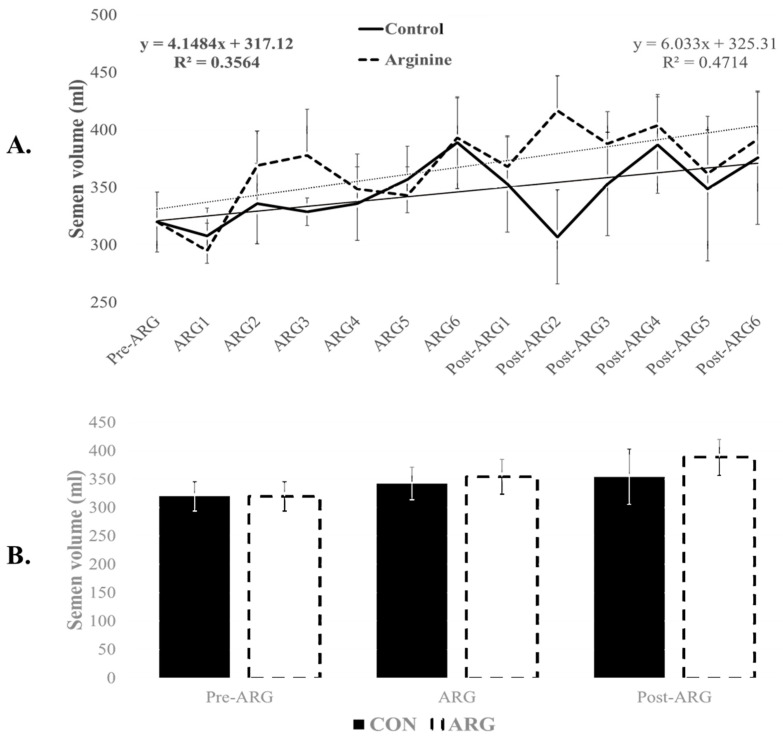
Effect of dietary arginine supplementation on semen volume. Boar semen was collected two weeks before (Pre-ARG), then for six weeks during arginine feeding (ARG), and six weeks after arginine feeding (Post-ARG). Weekly (**A**) and period–timepoint (**B**) semen data (mean ± sem) are provided. No significant differences (*p* > 0.05) were observed.

One boar was excluded from the CON group due to sickness and consistently lower semen quality. Before the feeding trial, the semen volume from the nine boars averaged 320 ± 26 mL. This production increased progressively during the feeding trial, reaching 354 ± 40 mL (R^2^ = 0.356) in the CON group and 389 ± 40 mL (R^2^ = 0.471) in the ARG group at the end of the trial (Figure 1A). Although the ARG group had numerically higher volumes, immediately after the arginine feeding, both groups were not significantly different (CON: 343 ± 29 mL and ARG: 355 ± 31 mL; *p* > 0.05; Figure 1B). Similarly, sperm concentrations (million/mL) were not significantly different between CON and ARG during arginine (130 ± 20 and 140 ± 10, respectively) and post-arginine feeding (130 ± 10 and 140 ± 10, respectively).

Overall, arginine-fed (ARG) boars produced non-significantly higher semen volume and sperm concentration than the control group (368 ± 34 mL and 140 ± 10 million sperm/mL versus 346 ± 31 mL and 130 ± 20 million sperm/mL; *p* > 0.05). Similarly, total sperm production did not change significantly over the three-period feeding trial (*p* > 0.05), although the ARG group maintained consistently higher mean numbers than their CON counterparts (*p* > 0.05). Indeed, with an overall sperm count of 47 ± 4 × 10^9^ in the pre-ARG period, this number increased to 49 ± 6 × 10^9^ after arginine supplementation (ARG) versus 42 ± 7 × 10^9^ in the CON, and to 55 ± 6 × 109 at the end of the trial (post-ARG) versus 45 ± 8 × 10^9^ in the CON. There was no significant difference between the two groups at any time (*p* > 0.05). However, boars on arginine treatment produced a more substantial number of sperm throughout the feeding trial (ARG and post-ARG) than the CON group (52.0 ± 5.8 × 10^9^ versus 44 ± 5 × 10^9^; *p* > 0.05). Total sperm production resulted in an average of 21 ± 3 semen doses in the CON group versus 24 ± 2 in the ARG group (*p* = 0.05), indicating a potential treatment effect on semen doses.

**Figure 2:**
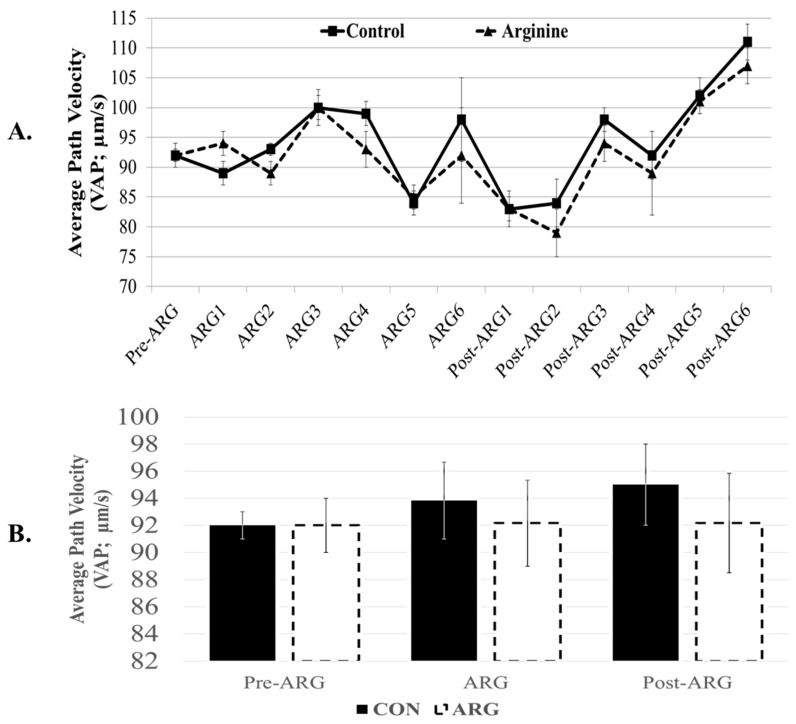
Effect of dietary arginine supplementation on Velocity of Average Path (VAP) of spermatozoa. Boar semen was collected two weeks before (Pre-ARG), then for six weeks during arginine feeding (ARG), and six weeks after arginine supplementation (Post-ARG). Semen was collected twice weekly from those control (CON, *n* = 4) and arginine-supplemented (ARG, *n* = 5) boars. Weekly (**A**) and period–timepoint (**B**) semen data (mean ± sem) are provided. Spermatozoa were analyzed with the Hamilton Thorne CASA set at 37 °C. There were no significant differences among data (repeated-measurement ANOVA-2 (in **A**) and ANOVA-2 (in **B**), followed by student’s *t*-test).

We have not observed any significant effects of L-arginine supplementation on total and progressive motility of spermatozoa. The overall effects of arginine were 81 ± 3% and 64 ± 5% in the ARG group vs. 81 ± 3% and 66 ± 4% in the CON group, respectively (*p* > 0.05). Similarly, numerous examined kinematic parameters (i.e., VCL, VSL, VAP, amplitude lateral head displacement or ALH, and beat cross frequency or BCF) and abnormal morphology sperm defects (bent tails, coiled tails, proximal droplets, and distal droplets) were not significantly (*p* > 0.05) affected by the dietary arginine supplementation of L-arginine (Table 1). Additionally, the straightness of the sperm path (linearity or LIN; VSL/VCLx100), the righteousness of sperm motion (Straightness or STR; VSL/VAPx100), and the degree of oscillation of the sperm head to VAP (balancing or wobble, WOB; VAP/VCLx100) were not significantly affected by the arginine supplementation (*p* > 0.05; Table 1). Figure 2 depicts the non-significant (*p* < 0.05) variations of VAP either weekly (A) or after combining data in individual period-–timepoint groups (B).

### 3.2. Proteomic Analyses and Identification of Differentially Expressed Proteins

**Figure 3:**
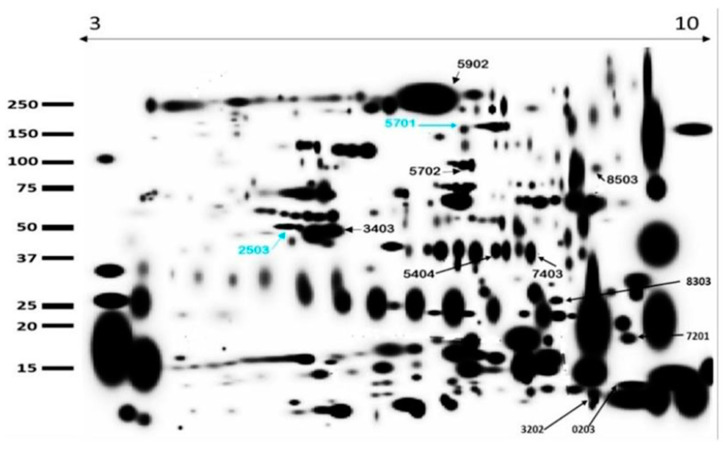
Representative gel electrophoresis of porcine seminal plasma proteome. This merged gel electrophoresis of CON (*n* = 3) and ARG (*n* = 3) groups revealed two downregulated (blue arrows) and ten upregulated (black arrows) protein spots by dietary arginine supplementation.

Figure 3 is a combined representative gel electrophoresis of seminal plasma derived from the control (CON) and arginine (ARG) groups. The image matches three independent and highly reproducible gel replicates, for which qualitative analyses indicated averages of 322 and 317 in the CON and ARG groups, respectively, with 12 spots appearing significantly differentially expressed (*p* < 0.05; Figure 3).

**Figure 4:**
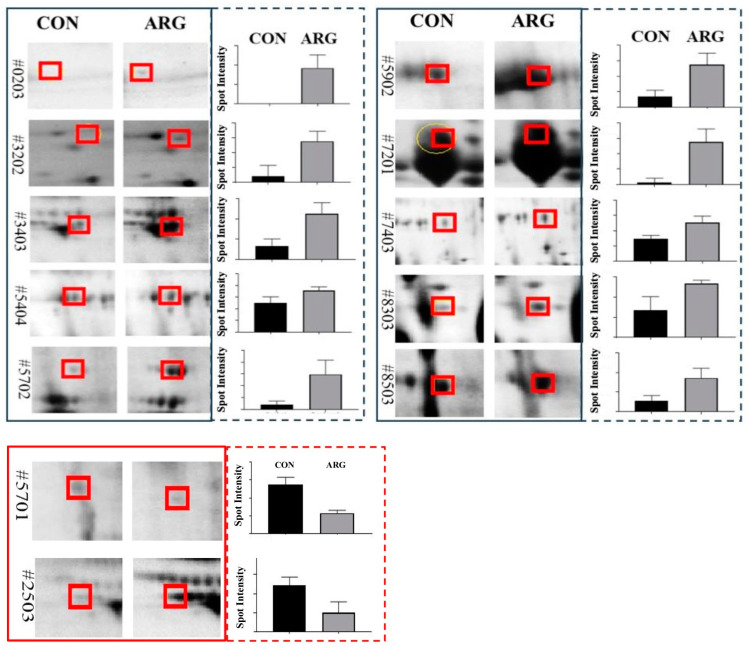
Effect of dietary L-arginine supplementation on boar seminal plasma protein expression. Solid black and red boxes represent up- and down-regulated protein spots. Quantified intensities of protein spots are shown in corresponding dotted colored boxes.

Ten spots (0203, 3202, 3403, 5404, 5702, 5902, 7201, 7403, 8303, and 8503) were up-regulated in the ARG group and identified as porcine seminal protein II (PSP-II), porcine seminal protein I (PSP-I), fibronectin 1 (FN1), glutaminyl-peptide cyclotransferase (QPCT), cathepsin B (CTSB), TIMP metallopeptidase inhibitor 2 (TIMP2), lactotransferrin (LTF), serpin family I member 1 (SERPINI1), complement factor H (CFH), and zymogen granule protein 16 homolog B-like (LOC110259943). In contrast, two protein spots (5701 and 2503) were downregulated and were identified as angiotensin I converting enzyme (peptidyl-dipeptidase A) 1 (ACE1) and keratin, type I cytoskeletal 14 (LOC110255312). Quantitative analyses of protein spots revealed 1.6- to 100-fold upregulation and downregulation of 0.32 (−3.1) to 0.36 (−2.8) fold changes (Figure 4 and Table 2).

Table 2 summarizes the characteristics of the twelve differentially expressed proteins. All these proteins showed a 100% protein score confidence interval (C.I.) % and total ion C.I. %. Functional analyses of the upregulated proteins indicated significant enrichment of the “Extracellular region” (GO:0005576; FDR = 10^−6^) and “Extracellular space” (GO:0005615; FDR = 0.0037), associated with a cellular component. Protein-to-protein interactions (PPI) showed a non-significant (*p* = 0.1) interaction between FN1 and TIMP2 related to GO:0002020~Protease binding. There was a significant enrichment in the keyword “Disulfide bond” (FDR = 0.0407), annotated KW-1015, including for proteins like FN1, TIMP2, PSP-I, PSP-II, CFH, CTSB, LTF, QPCT, and ZGP16B. Additionally, the “Degradation of the extracellular matrix” Reactome pathway (SSC-1474228) was significantly enriched in various proteins, including FN1, CTSB, and TIMP2. There was no significant enrichment found with the downregulated proteins (ACE1 and KRT14).

## 4. Discussion

This study highlights the essential role of dietary L-arginine supplementation in improving reproductive performance. An innovative aspect is its impact on boar semen production and its seminal plasma proteome.

Over a six-week feeding trial, the research ensured consistent exposure of spermatozoa to L-arginine during all phases of spermatogenesis, right up to ejaculation. Dietary arginine supplementation did not result in significant changes in semen volume, or the total number of spermatozoa produced, and the sperm quality—such as motility, kinematics, and morphology—remained unaffected. These results are consistent with prior studies demonstrating the benefits of L-arginine in deficient or various physiological conditions. For instance, beneficial effects of L-arginine have been reported in (1) improving semen quality and libido in summer heat-stressed boars [12], (2) boosting testosterone levels and improving semen quality and spermatogenesis in older roosters [31], (3) enhancing sperm counts and motility in men with oligospermia [32,33] and enhancing motility in vitro [34], and (4) facilitating capacitation and acrosome reactions of cryopreserved bovine spermatozoa [35]. In a boar study conducted under stress or inadequate arginine conditions, the authors reported an effectiveness of 0.8% and 1.0% of dietary L-arginine supplementation [12]. In our study, the control diet containing 0.77% arginine was supplemented with 1.0%, totaling 1.77% in the treatment diet. Considering that the optimal amount of arginine in boar diets remains undetermined, the observed data may highlight a limited effect of high doses of arginine on sperm motility. Notably, the increases in semen volume, total spermatozoa, and the number of semen doses suggest tremendous potential for artificial insemination doses, providing significant benefits for breeders in enhancing reproductive efficiency and fertilization success.

Although arginine supplementation barely affected sperm quality in our study, its role in modulating the seminal plasma proteome, as revealed by omics technology, provides further insights into enhancing spermatozoa functional capacity [36]. Studies have described several seminal plasma proteins for accurate, precise, and comprehensive evaluation of boar reproductive health compared to conventional spermatozoa characteristics, making those proteins valuable markers in predicting sperm quality and boar fertility [37,38,39]. A recent study reported higher farrowing rates and litter size from seminal protein-selected boars [40], potentially impacting pig productivity. Previous research showed the significant impact of dietary arginine on seminal plasma, mainly through bioassays measuring markers like malondialdehyde and antioxidative enzymes [12]. However, large-scale studies on arginine’s effects on the seminal plasma proteome are lacking, and there is a need for further exploration in this critical area of reproductive health. The gel-based proteome analysis conducted in this study identified 12 differentially expressed proteins, and the bioinformatic assessments suggest that these differential expressions may enhance sperm fertility. Identifying seminal plasma proteins as biomarkers for boar fertility is a significant step in reproduction science [30,37]. However, research on dietary impacts on the proteomic profile of boar seminal plasma is limited.

Our study showed that two proteins, angiotensin I-converting enzyme 1 (ACE1) and Keratin, type I cytoskeletal 14 (KRT14), were downregulated in boars on an L-arginine-supplemented diet. Angiotensin-converting enzyme (ACE) in seminal plasma catalyzes the formation of angiotensin II. It binds to receptors on sperm, with its presence in seminal plasma associated with sperm density, motility, viability, and fertilization in various livestock species [23]. Although ACE1 is associated with enhanced sperm motility and function, higher nitric oxide (NO) levels because of arginine supplementation could lead to the downregulation of seminal ACE1 due to cellular feedback mechanisms or altered enzymatic pathways, which could be associated with sperm DNA damage [41,42]. Moreover, the ACE’s testicular and seminal plasma origins differentially affect sperm function [24]. The testicular isoform in spermatozoa is essential for sperm–egg fusion through A Disintegrin and Metalloprotease 3 (ADAM 3) [43,44]. In contrast, the ACE activity of the seminal plasma isoform varies with sperm concentration and may not be associated with sperm function [24]. KRT14, while known for supporting epithelial cell structure, may also influence sperm motility [45,46], although its direct role in seminal plasma has yet to be established. In both cases, the bioinformatics analysis did not reveal any clue to protein downregulation, leaving more research to shed light on their effect on boar fertility. However, the downregulation of KRT14 might indicate cellular disruption, potentially compromising the structural stability of the seminal plasma environment, which is essential for protecting sperm during ejaculation and storage [47]. These downregulations highlight the requirement for the correct dose of dietary arginine to avoid possible detrimental effects.

On the other hand, the ten significantly upregulated proteins in the seminal plasma of boars receiving L-arginine supplementation were those proteins—fibronectin 1 (FN1), tissue inhibitor of metalloproteinase 2 (TIMP2), porcine seminal proteins I and II (PSP-I and PSP-II), complement factor H (CFH), cathepsin B (CTSB), lactotransferrin (LTF), glutaminyl-peptide cyclotransferase (QPCT), zymogen granule protein 16 homolog B-like (ZGP16B), and neuroserpin (SERPINI1)—that play vital roles in male fertility.

Notably, FN1 and TIMP2 interact with each other and co-expressed in crucial cellular physiological processes [48,49]. FN1 is essential for sperm capacitation and fertilization [50,51] and may serve as a biomarker for sperm selection in IVF. The synergistic relationship between FN1 and TIMP2 could significantly enhance semen quality and male fertility. TIMP2 inhibits metalloproteinases (MMPs) 2 and 9, facilitating the remodeling of the extracellular matrix and improving semen liquefaction [52]. Research shows that humans with lower levels of TIMP2 in their seminal plasma exhibit higher sperm DNA fragmentation than those with higher TIMP2 levels [53], indicating its protective role in safeguarding sperm DNA integrity. Additionally, reduced TIMP2 is linked to inflammatory responses that harm sperm function and integrity. Hence, the co-increase in FN1 and TIMPs underscores their critical role in maintaining semen quality and protecting sperm from damage, emphasizing the potential for advancements in male reproductive health. Porcine seminal fluid is vital for male fertility due to the PSP-I/PSP-II complex comprising glycosylated spermadhesins [54]. This heterodimer complex protects spermatozoa by modulating the immune response in the female reproductive tract, ensuring sperm survival and function after ejaculation and deposition [54,55]. Dietary L-arginine supplementation can enhance PSP-II’s binding to the zona pellucida, improving fertilization success. Complement factor H (CFH) protects sperm from immune attacks by preventing anti-sperm antibody formation in males, thus supporting fertility [56]. Cysteine protease B (CTSB) is crucial for spermatogenesis and sperm maturation, regulating autophagy and apoptosis [57]. Present in various biofluids, including semen [58], CTSB’s absence can lead to decreased sperm counts and motility and increased morphological abnormal sperm [57]. Lactotransferrin (also known as lactoferrin) contributes to the antimicrobial defense system [59], and its interaction with the epididymal protease inhibitor (EPPIN) present on the surface of spermatozoa, thereby regulating sperm motility and function [60]. These proteins, among others, play a crucial role in protecting sperm from infections and facilitating their effective functioning within the female reproductive tract, ultimately contributing to successful fertilization [59].

Altogether, the interplay of these upregulated proteins highlights the intricate mechanisms supporting male reproductive health and fertility under dietary L-arginine supplementation. However, there is a need for more research to understand the specific roles and mechanisms of some identified upregulated proteins in our study, including QPCT, ZGP16B, and SERPINI1. For instance, SERPINI1 is a neuroserpin serine protease inhibitor primarily known for its role in the nervous system and regulating axonal growth and synaptic plasticity [61,62]. Although we do not fully understand its role in semen physiology and male reproductive health, its regulatory role in enzymatic activities can likely influence sperm function and fertility. Additionally, investigating the effects of dietary arginine supplementation on the lipid composition of seminal plasma could provide valuable insights into the mechanisms that modulate membrane fluidity and sperm motility [63], influencing fertility.

## 5. Conclusions

A key finding of this study is that dietary arginine supplementation (up to 1.77%) modified the proteome of seminal plasma without affecting routine sperm quality parameters. This finding suggests that arginine may influence seminal proteins, potentially improving seminal plasma quality and sperm biology, with promising implications for boar fertility. Despite the downregulation of ACE1 and KRT14, which may indicate inhibitory effects of arginine supplementation on critical proteins required for boar reproductive health, these insights pave the way for future research on the impact of dietary arginine supplementation on sperm DNA packaging and overall boar fertility and determining the optimal arginine supplementation requirement.

## Figures and Tables

**Table 1:** Effects of dietary L-arginine supplementation on some boar spermatozoa parameters.

Parameters	Pre-ARG	ARG		Post-ARG		Overall	
		Control	Arginine	Control	Arginine	Control	Arginine
**TM (×10^9^)**	81 ± 2	81 ± 3	81 ± 4	81 ± 2	82 ± 3	81 ± 3	81 ± 3
**PROG (%)**	71 ± 3.5	67 ± 4	64 ± 5	64 ± 4	64 ± 4	66 ± 4	64 ± 5
**VSL (μm/s)**	68.5 ± 1.5	63 ± 3	58 ± 3	63 ± 3	58 ± 3	64 ± 3	59 ± 2
**VCL (μm/s)**	186 ± 7	190 ± 8	192 ± 9	194 ± 9	194 ± 10	192 ± 8	192 ± 9
**VAP (μm/s)**	91 ± 1	94 ± 2	92 ± 3	95 ± 3	92 ± 4	94 ± 3	92 ± 3
**LIN (%)**	38.4 ± 1.5	35.3 ± 0.2	33 ± 0.1	35 ± 0.3	33 ± 0.3	35 ± 0.6	32.5 ± 0.7
**STR (%)**	73.2 ± 0.3	69 ± 0.3	65 ± 0.2	69 ± 0.5	66 ± 0.2	68 ± 1	65 ± 1
**WOB (%)**	51 ± 1	50 ± 0.1	49 ± 0.1	50 ± 0.1	49 ± 0.3	50 ± 0.3	49 ± 0.5
**BCF (Hz)**	39 ± 0.7	38 ± 1	38 ± 1	38 ± 1	37 ± 1	38 ± 1	38 ± 1
**ABNOR (%)**	3.6 ± 0.75	3 ± 1	3 ± 1	3 ± 1	3 ± 1	3 ± 1	3.2 ± 1
		*p* > 0.05		*p* > 0.05		*p* > 0.05	

TM: total motility; PROG: progressive; VSL: straight-line velocity; VCL: curvilinear velocity; VAP: velocity of the average path; LIN: linearity; STR: straightness; WOB: wobble; BCF: beat cross frequency. ABNOR: abnormalities = percentage of sperm with defects (bent tail, coiled tail, distal droplet, proximal droplet). *p* > 0.05: no significant difference.

**Table 2:** Twelve differentially expressed proteins upon dietary arginine supplementation.

Differentially Expressed Proteins in the Arginine Group							
Expression Direction	Protein Spot # Numbers	MW (kDa)	PI	NCBI-ID	Protein Name	FC *	Biological Function
Up	0203	14.8	8.7	NP_999001	Porcine seminal protein II (PSP-II)	100×	Extracellular
	3202	14.5	8.1	NP_999002	Porcine seminal protein I (PSP-I)	11×	Extracellular
	5902	288.8	6.0	XP_003133690	Fibronectin 1 (FN1)	5.6×	Binding
	7403	41.1	6.8	XP_003481293	Glutaminyl-peptide cyclotransferase (QPCT)	2.2×	Catalytic activity
	5404	43	6.7	XP_005657322	Cathepsin B (CTSB)	1.6×	Catalytic activity
	8303	27	8.2	XP_020921860	TIMP metallopeptidase inhibitor 2 (TIMP2)	1.7×	Enzyme regulator activity
	8503	77.6	8.2	XP_020924222	Lactotransferrin (LTF)	4×	Catalytic activity;enzyme regulator activity; metal ion binding
	3403	46.4	4.9	XP_020925421	Serpin family I member 1 (SERPINI1)	4.2×	Enzyme regulator activity
	5702	138.5	6.6	XP_020937501	Complement factor H (CFH)	6×	Binding; regulatory activity
	7201	18.2	8.7	XP_020942404	Zymogen granule protein 16 homolog B-like (LOC110259943)	28×	Regulatory activity
Down	5701	150.3	6.6	NP_001077410	Angiotensin I converting enzyme (peptidyl-dipeptidase A) 1 (ACE)	0.4×	Catalytic activity; metal ion binding
	2503	51.6	5.2	XP_020922659	Keratin, type I cytoskeletal 14 (LOC110255312)	0.3×	Structural activity

MW = molecular weight; PI = Isoelectric Point (PI); * FC = fold change.

## Data Availability

All generated data are provided in this submission.
